# Tumor Growth, Proliferation and Diffusion in Osteosarcoma

**DOI:** 10.1007/s10441-025-09494-4

**Published:** 2025-03-18

**Authors:** M. I. Romero Rodríguez, J. C. Vargas Pino, E. L. Sierra-Ballén

**Affiliations:** 1 Departamento de matemáticas. Facultad de Ciencias Básicas y Aplicadas, Universidad Militar Nueva Granada, Km 7 Cajicá-Zipaquirá, Cajicá, Cundinamarca 250240 Colombia; 2 Ingeniería Biomédica. Facultad de Ingeniería., Universidad Militar Nueva Granada, Km 7 Cajicá_Zipaquirá, Cajicá, Cundinamarca 250240 Colombia; 3 Ingeniería Multimedia. Facultad de Ingeniería, Universidad Militar Nueva Granada, Km 7 Cajicá-Zipaquirá, Cajicá, Cundinamarca 250240 Colombia

**Keywords:** Osteosarcoma, Tumor growth, Power law, Logistic model, Diffusion

## Abstract

Osteosarcoma is the most common primary bone cancer. According to medical and biological studies, it has a high genetic complexity, thus, to differentiate the mechanisms of appearance and evolution of this disease is a difficult task. In this paper, we use three simplest and well known mathematical models to describe the behavior of several cell lines of osteosarcoma. First, we use a potential law to describe the tumor growth in immunosuppressed mice; with it we show that the variation of tumor growth has a sublinear behavior without the blow-up phenomenon. Second, the logistic model is used to obtain a good aproximation to the rates of proliferation in cell confluency in in vitro experiments. Third, we use a linear reaction-diffusion model; with it, we describe the diffusion behavior for some cell lines. These three models allow us to give a classification of cell lines according to the rates of tumor growth and proliferation and to the diffusion coefficient. A relationship is found between the rates of the tumor growth, the diffusion coefficient and tumorigenicity. Experimental data are extracted from Lauvrak et al. (British Journal of Cancer 109(8):2228–2236, 2013).

## Introduction

Osteosarcoma is one of the main types of bone cancer and it can present different degrees of malignancy; this disease represents from $20\%$ to $56\%$ of the diagnosed bone cancers (Gobin et al. 2014), it is usually very aggressive and it can cause early metastases (Bielack et al. 2002).

For the analysis of cancer in pre-clinical research, laboratories often use cell lines which come from cultures in which the cells have undergone adaptations to be able to proliferate in uncontrolled environments, allowing the description of underlying mechanisms of tumor progression.

According to medical and biological studies, osteosarcoma exhibits a high gene complexity (Schott et al. 2020), which makes the mechanisms of appearance and evolution of this condition to have a difficult explanation and a poor understanding.

The genetic complexity of osteosarcoma has been reflected in different clinical behaviors that each of the cell lines presents; an example of this situation is that some of them are highly metastatic and others are not, occasionally they exhibit wide variations with respect to the primary tumor, and many of them rapidly generate tumors while others have a poor tumor growth.

**Fig. 1:**
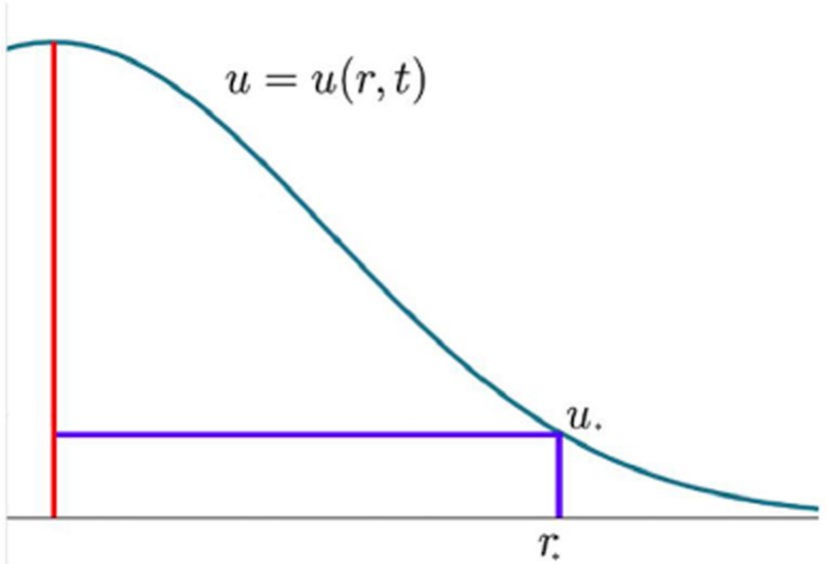
Cell density at time *t*

The first article that presented a generalized characterization of twenty-two osteosarcoma cell lines was Lauvrak et al. (2013); there, among other aspects, tumor growth was studied by means of in vivo experiments with immunosuppressed mice presenting a sublinear behavior [see Figs. 1 and 4 (Lauvrak et al. 2013)], a fact which is in agreement with what was stated in Pérez-García et al. (2020), where it is asserted that this type of behavior is inherent to 3d cultures. On the other hand, the experimental results obtained for in vitro studies of the cell confluence of each of the cell lines show a sigmoid behavior [see Supplementary Figs. S1 and S2 in Lauvrak et al. (2013)].

There exists a vast amount of literature concerning the mathematical modeling of cancer growth, see, for example (Rockne et al. 2019). Usually, ordinary differential equations of exponential type and different versions of the logistic model are used in order to determine the proliferative behavior of the cells. Since the last decades of the last century, there were proposed deterministic models in order to demonstrate the influence of diffusion and proliferation on tumor growth (Murray 2003). There, the diffusion is related to the active motility of glioblastoma cells. Other reaction-diffusion models were used in order to describe the spacial distribution of tumor cells for different moments of time (see Jiang et al. (2014) and references therein). In Le et al. (2021), for example, there are presented mathematical models in order to analyze the impact of the immune cell interactions on the growth of osteosarcoma tumors that have distinct immune patterns. In Colson et al. (2021) a system of coupled partial differential equations is studied; this system couples the density of the tumor cells to that of healthy cells and describes the fact that tumors use biological strategies such as the Warburg effect in order to destroy the extracellular matrix and thus to invade the healthy tissue. In Barros et al. (2021), a mathematical platform was developed in order to enable in-silico experiments and to investigate the interplay between tumor cells, effector cells, and memory CAR-T cells in immunodeficient mouse models of hematological cancers. In Hu et al. (2022) the authors propose, as a model of osteosarcoma growth, a system of reaction-diffusion-advection equations coupled to the Biot equations of poroelasticity, thus exploring the effects of infiltration of immune cells in the tumoral tissue. However, as far as we know, there are no studies which apply the simplest mathematical models to the description of spatio-temporal behavior of specific lines of osteosarcoma reported in Lauvrak et al. (2013).

Mathematics in oncology could be approached in two manners: on the one hand, mathematical models can be used to answer specific questions about cancer. On the other hand, the proper biological processes associated with cancer could be the inspiration to develop new mathematical theories. Both approaches are valuable but may be limited by the lack of access to detailed data on cancer biology (Brady and Enderling 2019).

In this article we give a simple mathematical description of three topics related to the evolution of cancerous tumors: growth of tumor volume, proliferation, and diffusion. Each of these aspects is analyzed for different osteosarcoma cell lines using either ordinary or partial differential equations.

Tumor growth is defined as the change in tumor volume over time. Cell proliferation measures to which extent the cells of a given cell line are able to cover the area of a Petri dish. Here, we assume that tumor growth is also related to diffusive processes due to the existence of chemotactic processes.

Using the method of least squares, the experimental data were fitted to the parameters of equations used to describe tumor growth and cell proliferation.

To obtain our numerical results, we decided to use only the data reported in Lauvrak et al. (2013) in order to guarantee unification in laboratory practices and reliability in measurements.

For the description of tumor growth, the potential model (see (1) below) was used for the calculation of the values of the growth rate $\alpha$ and the scale exponent $\beta$. From the numerical results, it can be observed that the model fits well with the experimental data for tumorigenicity (Section 3.1). Tumorigenicity refers to the ability of tumor cells to form new tumors.

For the description of cell proliferation, the logistic model (3) was used, and the cell proliferation rates $\rho$ of the in vitro experiments in the absence of treatments were calculated.The model used fits sufficiently well to the experimental data. An interesting fact is the appearance of significant changes according to the quantity of cells planted, either at $5\%$ or $10\%$ of the corresponding Petri dish area, suggesting varying degrees of sensitivity due to the passage number (Sect. 3.2).

**Fig. 2:**
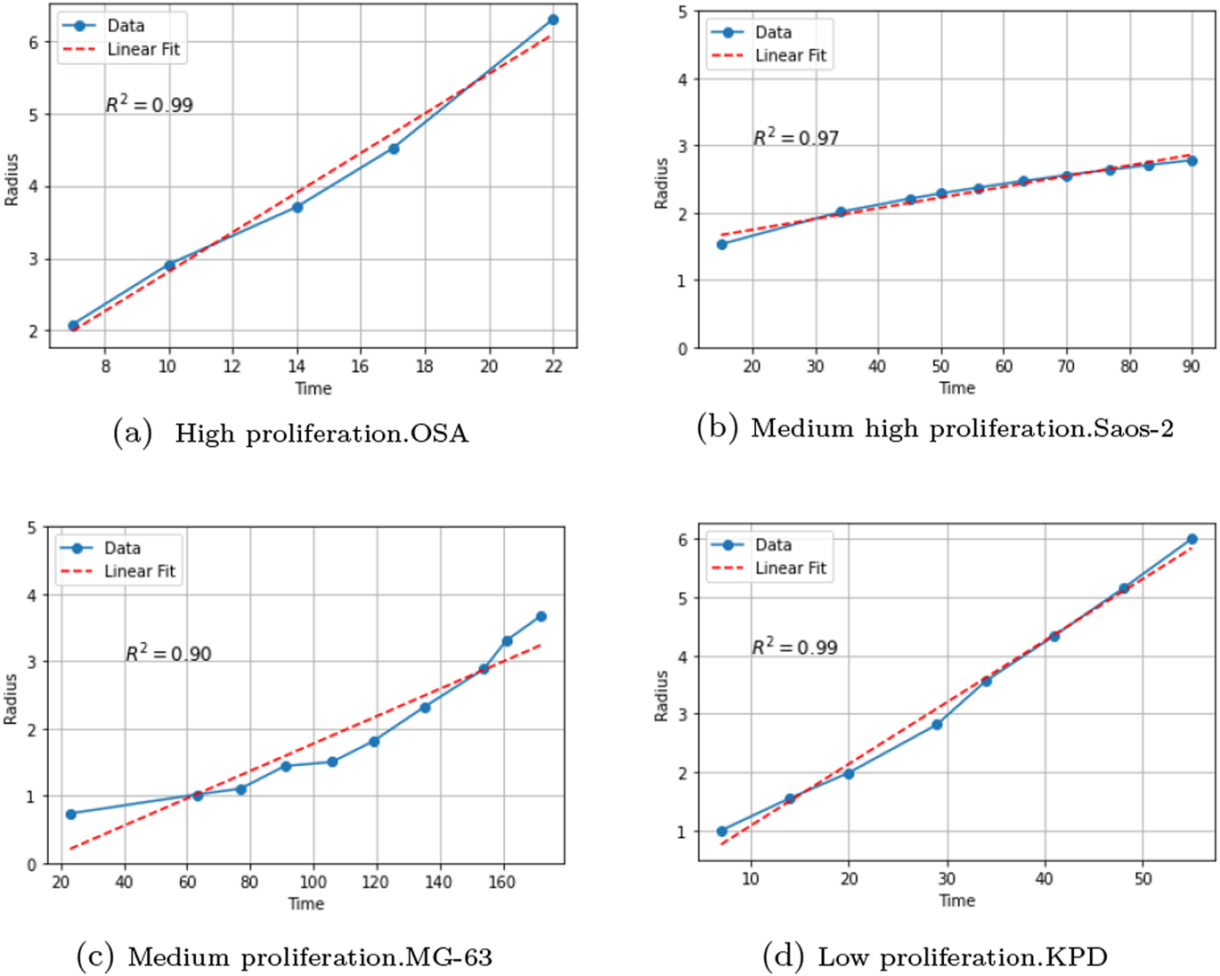
Radius evolution in time for some cell lines with different levels of proliferation. Data obtained from Lauvrak et al. (2013) (supplementary Fig. S1)

The model that we use to describe diffusion corresponds to the problem given in (5) (see Sect. 2.4). The geometry considered in this section is based on the biological hypothesis that tumors tend to grow centrifugally with the configuration of spheres (Greenspan 1972; Sutherland 1988). From the experimental data for tumor volume, it was observed that the radii appeared to evolve linearly in time (see Sect. 2.4 and Fig. 2) and hence the diffusion coefficient ${\bar{D}}$ tends to remain close to a constant value (see Sect. 3.3 and Fig. 6).

Upon conducting a correlation study between numerically calculated parameters ($\alpha$, $\beta$, $\rho$, and ${\bar{D}}$) and experimental data, a strong correlation was found between the growth rate classification $\alpha$ and tumorigenicity. Additionally, a strong correlation was observed between tumorigenicity and the diffusion coefficients ${\bar{D}}$ (Sect. 3.4).

All the scenarios mentioned above are of course the simplest and well-known mathematical models frequently used in biomathematical studies of cancer growth in research about mathematical oncology. These models were selected because they utilize a limited number of parameters, which can be estimated with a modest amount of data. Consequently, they enable the extraction of meaningful biological insights.

## Cell Lines and Models

In this paper, three mathematical models are presented; these allow us to describe the evolution of some osteosarcoma cell lines using data from experiments reported in Lauvrak et al. (2013) in immunosuppressed mice and for cell proliferations in in vitro experiments.

The first model consists of an ordinary nonlinear differential equation that describes the change in tumor volume, which allows us to determine the volume variation rates and the scaling exponent to describe the power law that governs each cell line.

The second model corresponds to the logistic model. Here the experimental data of cell proliferation were adjusted to determine the proliferation rates from in vitro studies.

The third model corresponds to a linearization of the Fisher-Kolmogorov equation which allows us to describe the behavior of diffusion. We use the values of the cell proliferation rates obtained from the logistic model and the tumor volume experimental data.

Using the well-known fact that initially solid tumors grow centrally tending to form sphere-like configurations (although in more advanced stages the shapes of the tumors vary according to the affected tissue), we will assume that the tumors maintain this shape during the total growth process; this condition allows us to make approximate calculations for tumor growth according to (1) and for diffusion using problem (5).

To calculate the values of the parameters in models (1) and (2), the method of least squares was employed using algorithm implementations in Python. This approach resulted in approximations to the experimental data with relatively high values of the coefficient of determination $R^2$.

### Experimental Data and Cell Lines

This study was based on experimental data obtained in Lauvrak et al. (2013); to analyze the behavior of tumor growth, we selected in vivo data from the Supplementary Figure S1, corresponding to the cell lines HOS, OHS, OSA, HOS-143B, HOS-MNNG, MHM, HAL, IOR/OS9, ZK-58, Cal-72, Saos-2, G-292, KPD, IOR/OS14, IOR/OS15, U2OS and MG-63. The tumor volumes were measured in $\hbox {mm}^3$ and their evolution times were measured in days (Lauvrak et al. 2013).

To analyze the behavior of the cell proliferation we used in vitro data obtained from Lauvrak et al. (2013), Supplementary Figure S2. Proliferation rates as cell confluence $(\%)$ over time (*h*) are presented there. The original experiments were cultured with initial conditions at $5\%$ and $10\%$ of cell confluence. In this part, the cell lines analyzed in our study were OSA, MHM, U2OS, HOS-143B, IOR/OS15, IOR/OS10, CAL-72, Saos-2, IOR/OS18, HOS, OHS, IOR/SARG, MG-63, HOS/MNNG, ZK-58, IOR/OS9, IOR/OS14, HAL, KPD, IOR/MOS, G-292. For our approximation we adjust the scale of the time data to days to have the same scale as in the in vivo data.

Under the assumption of spherical solid growth, the tumor radii were calculated for experimental tumor volume from the Supplementary Figure S1. These data, along with the previously calculated value of the logistic parameters $\rho$, were used to determine the diffusive behavior of each of the cell line studied.

### In Vivo Tumor Growth. Power Laws

Different biochemical studies have shown that metabolic alteration and high glucose uptake promote a malignant tumor growth (Dang and Semenza 1999). Usually, cancer cells present the Warburg effect; in Pérez-García et al. (2020) the authors state that malignant tumors scale between the metabolic requirements of combined tissues governed by the principle of minimum energy and the metabolic requirements of independent uncoordinated units, observing that, in general, the total size of the injury is proportional to metabolic tumor volume. In Altenberg and Greulich (2004), cancers are classified according to the overexpression of glycolysis genes, and it is stated that cancers such as those of lymph nodes, prostate and brain always overexpress such genes while cartilage and bone marrow cancers only present its alteration sporadically. Nevertheless, the effect of glycolysis on the growth of osteosarcoma tumors is still poorly understood.

In this paper we will assume that the Warburg effect occurs in osteosarcoma cell lines promoting tumor growth in immunosuppressed mice, which allows us to assume that, according to allometric laws, the rate of variation of tumor volume over time is proportional to the volume growth at each moment. The corresponding well-known mathematical model (see, e.g., Pérez-García et al. (2020)) reads as follows:

1 $$\begin{aligned} \frac{dV}{dt}=\alpha V^\beta ,\quad V\mid _{t=t_0}=V_0,\quad V_0\ne 0, \end{aligned}$$

where $V=V(t)$ is the tumor volume, $\alpha$ is defined as a parameter that allows us to give a measure of the growth rate and $\beta \ne 0$ is the scale exponent. The values of $V_0$ are obtained from Lauvrak et al. (2013).

The solution of (1) is (we assume that $\beta \ne 1$):

2 $$\begin{aligned} V(t)=[V_0^{1-\beta }+\alpha (1-\beta ) (t-t_0)]^{\frac{1}{1-\beta }}. \end{aligned}$$

We assume that formula (2) is valid even when $V_0=0$ since at the start of the experiment the tumor volume is very small and imperceptible for its detection but of course is not zero. The last expression allows us to calculate the value of the parameters $\alpha$ and $\beta$, using the experimental volume data and curve fitting through the method of least squares. For this, we use a Python implementation of an unconstrained approximation for $\alpha$ and $\beta$ employing the Levenberg-Marquardt algorithm, with seeds $\alpha =0.9$ and $\beta =0.9$. This algorithm is implemented in optimize.curve$\_$fit^1^ of the Python library SciPy, for further information about SciPy see Virtanen et al. (2020).

The results obtained are discussed in Sect. 3.1 below.

### In Vitro Proliferation

In the study of the development of cancer tumors it is important to determine the specific cell line and its rate of proliferation to diagnose the stage of the disease with a high probability in order to identify the phase of the tumor and to make a prognosis.

To forecast the clinical behavior of a disease via the use of cell lines is a daunting task (Gillet et al. 2013). In osteosarcoma, the genetic complexity makes any description even more difficult; however, based on the data from the in vitro studies extracted from Supplementary Fig. S2 in Lauvrak et al. (2013), we will present some approximations of the cell proliferation rates which will help us to understand how aggressive is each one of the cell lines.

Since the experimental data for cell confluence have a sigmoid appearance, we will use the logistic model to calculate the proliferation rates measured in $1/\text {day}$. The model is given by:

3 $$\begin{aligned} \frac{dN}{dt}=\rho N\bigg (1-\frac{N}{k}\bigg ), \quad N\mid _{t=t_0}=N_0 \quad \text {with} \quad N_0 \ne 0, \end{aligned}$$

whose solution is

4 $$\begin{aligned} N(t)=\frac{kN_0e^{\rho t}}{k+N_0(e^{\rho t}-1)}. \end{aligned}$$

Here *N* represents the percentage of cell confluence, $N_0$ is the initial percentage and *k* is the load capacity, which we will assume to be equal to one hundred percent (as reported in the bibliographic data).

As in the previous section, the method of least squares was applied to the experimental data reported in Lauvrak et al. (2013) to obtain the values of the logistic parameter $\rho$ in the function described by (4). Such approximations are presented in Sect. 3.2 below.

### Diffusion Process

Cell diffusion is a phenomenon in which cell movement is guided by an extracellular chemical gradient, prompting tumor cells to migrate from the tumor’s origin point and to utilize extracellular matrix components to invade surrounding tissues. This process is believed to play a role in the invasion, extravasation, and metastasis of cancer cells (Roussos et al. 2011). In this context, understanding the role of diffusion in tumor biology becomes paramount for the development of effective therapeutic strategies to combat cancer.

Experimental findings from Lauvrak et al. (2013) provide insights into the approximate volume of tumors in in vivo data and the proliferation rates in in vitro data. Although cellular behaviors differ between these scenarios, combining the obtained data allows for the development of a model that qualitatively approximates the spatiotemporal behavior of osteosarcoma cell lines. In this model, we assume that tumors initially grow centrifugally and that cell reproduction follows an exponential growth pattern. The proposed model, inspired by (Murray 2003, p. 548) and Yin et al. (2019), serves as a framework for further understanding the dynamics of osteosarcoma progress and the influence of diffusion on tumor growth. Our (well-known) model is as follows:

5 $$\begin{aligned} u_t=D \Delta u+ \rho u\quad u\mid _{t=0}=C_0\delta (x), \end{aligned}$$

where $x\in \mathbb {R}^3$ is the position variable, *D* is the diffusion coefficient which will be calculated in this section, $\rho$ is the value of proliferation rate calculated in Sect. 2.3, $C_0$ is the approximation to the number of cells at the time the tumor was detected, $\delta (x)$ is the Dirac delta function and $u=u( x,t)$ is an exponentially decreasing function and represents the cell density at point *x* at time *t*. By the radial symmetry of (5), *u* depends only on the radial coordinate $r=\sqrt{x_1^2+x_2^2+x_3^2}$, that is, $u=u(r,t)$.

In model (5), it is assumed that the values of the coefficients *D* and $\rho$ remain constant. The well-known explicit solution to (5) is given by

6 $$\begin{aligned} u(r,t)= \frac{C_0}{8(\pi D t)^\frac{3}{2}}\exp \Bigg [\rho t-\frac{1}{4Dt}r^2\Bigg ]. \end{aligned}$$

The experimental data encompass various cell lines, each representing distinct types of lesions with considerable diversity in histologic features and grades, spanning different bone tissues (Klein and Siegal 2006). This inherent diversity leads to significant variability in the times of tumor appearance. According to the experimental findings, tumors may emerge anywhere between 3 and 8 days for cell lines exhibiting rapid tumor growth. For those with moderate growth rates, the onset of tumors occurs within a broader timeframe, ranging from 10 to 24 days. In contrast, cell lines characterized by slow growth exhibit even more extended intervals, with tumor appearance ranging from 34 to 78 days. This wide spectrum of tumor emergence times underscores the complexity of osteosarcoma progression and highlights the importance of considering various factors when studying its dynamics and developing therapeutic interventions.

As Fig. 1 shows, if the detectable density is $u_*$, then the radius $r_*$ of the tumor satisfies

$$\begin{aligned} u_*=\frac{C_0}{8(\pi D t)^\frac{3}{2}}\exp \Bigg [\rho t-\frac{1}{4Dt}r_*^2\Bigg ] \end{aligned}$$

and hence is given by

7 $$\begin{aligned} r_*^2=4D\rho t^2-4Dt\ln \bigg (\frac{8u_*}{C_0}(\pi Dt)^{3/2}\bigg ). \end{aligned}$$

Since the numerical values of $u_*$ and $C_0$ are unknown, the only way to use (7) is to assume, as in equation (11.14) in (Murray 2003, p. 548) (see also Mandonnet et al. (2008)) that the time *t* in (7) is sufficiently large in order to neglect the second term in (7) (of order $t\ln {t}$) in comparison with the first term (of order $t^2$). Thus, approximately,

8 $$\begin{aligned} r_*^2\approx 4D\rho t^2. \end{aligned}$$

The last formula means that the tumor radius evolves linearly in time. This behavior is confirmed by the graphs of the radius evolution taken from Lauvrak et al. (2013) and the linear fits for some cell lines shown in Fig. 2. All the other cell lines present a similar behavior.

Note that (8) implies that the velocity of propagation of the tumor $v=r_*/t=2\sqrt{D\rho }$ coincides with the velocity of the wave front predicted by the nonlinear Kolmogorov-Fisher equation.

Finally, equation (8) defines the diffusion coefficient via the values of the tumor radius $r_*$ and the time *t*:

9 $$\begin{aligned} D\approx \frac{r_*^2}{4\rho t^2}. \end{aligned}$$

The results obtained are discussed in Sect. 3.3 below. As we will see, our approximate formula (9) for the diffusion coefficient is consistent with the experimental data for most of the cell lines (see Sect. 3.4), that is, the cell lines that exhibit greater tumorigenicity and greater growth rate of the tumor also exhibit greater diffusion coefficients.

## Results and Analysis

### In Vivo Tumor Growth. Power Law

In this section we present the results obtained according to the model given in Sect. 2.2. In this model tumor growth was described as a power law problem. Our goal is to obtain a good curve fitting searching for the best parameters $\alpha$ and $\beta$ in the Solution (2). To start the fitting algorithm we fixed the seeds $\alpha =0.9$ and $\beta =0.9$, the results obtained are shown in Table 1.

**Table 1:** Approximation of $\alpha$ and $\beta$ parameters for power law

	Cell line	$\alpha$	$\beta$	$R^2$
High	HOS	2.1797	0.5300	99.79%
	OHS	1.1640	0.6696	99.00%
	OSA	1.1353	0.7198	99.61%
	HOS-143B	0.8803	0.8649	99.99%
	HOS-MNNG	0.8598	0.8555	99.99%
	MHM	0.8137	0.8633	99.99%
Medium high	HAL	0.4663	0.8530	98.92%
	IOR/OS9	0.4351	0.8134	99.09%
	ZK-58	0.3742	0.8781	99.80%
	Cal-72	0.3021	0.8562	99.67%
Medium	Saos-2	0.2573	0.7161	99.38%
	G-292	0.2016	0.8619	99.14%
	KPD	0.1668	0.8704	99.82%
	IOR/OS14	0.1576	0.7687	96.66%
Low	IOR/OS15	0.1251	0.6742	99.69%
	U2OS	0.1000	0.8419	99.15%
	MG-63	0.0801	0.8377	99.53%

Ordered from greater to smaller according to the values of $\alpha$. $R^2$ is the coefficient of determination obtained in the curve fitting

**Fig. 3:**
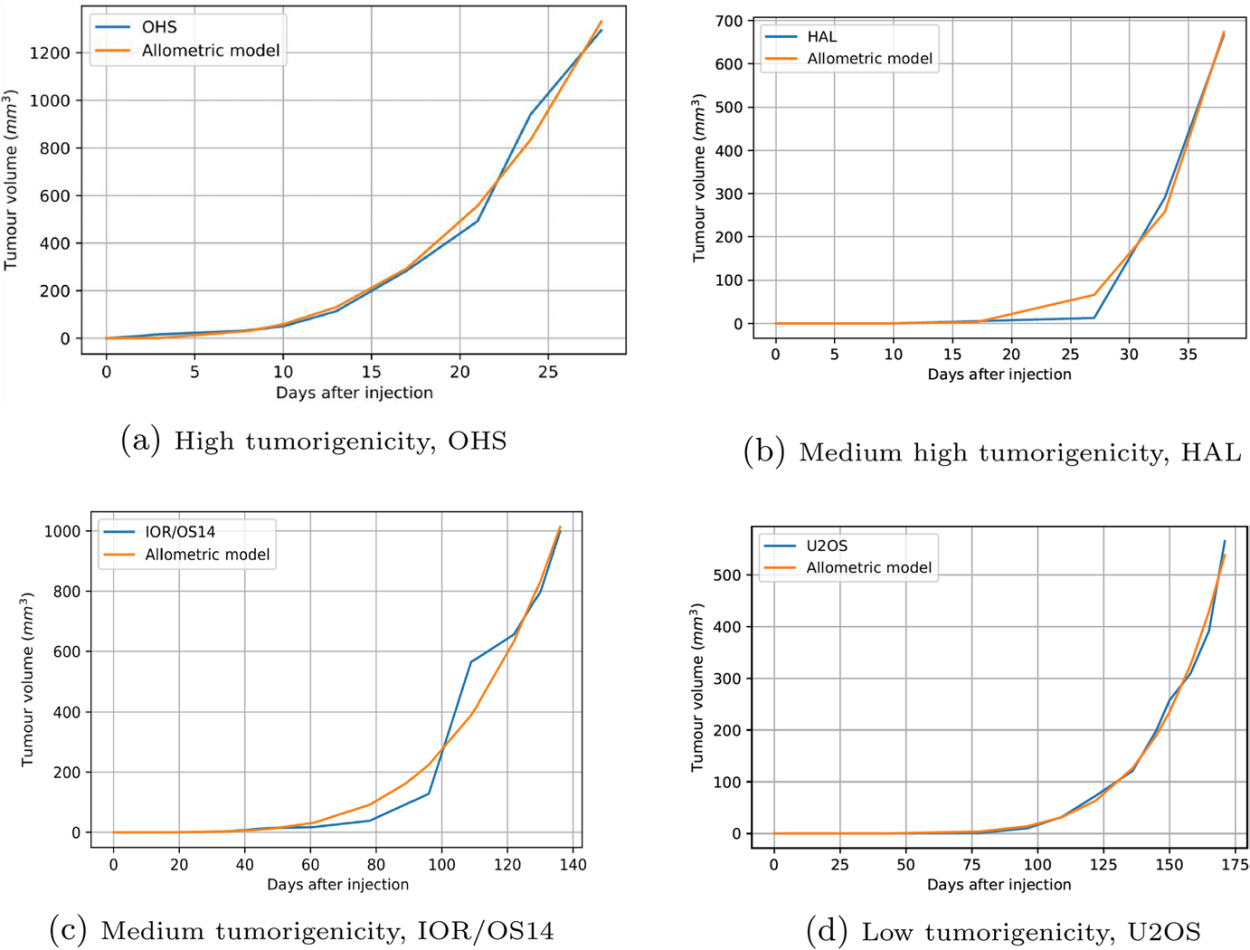
Power law approximations in the cell line with the lowest $R^2$ for each of the subgroups High, Medium High, Medium and Low. Curves in light blue color correspond to original line cell data obtained from Lauvrak et al. (2013) (supplementary Fig. S1). The values $t_0=0$, $V_0=0$ were taken

The $\alpha$ values allow us to rank cell lines from high to low according to their rate of change in tumor growth. The results obtained here are similar to those obtained experimentally with coefficients of determination $R^2>0.9$. Some examples of power law approximations and experimental data are shown in Fig. 3; the graphs for other cell lines are similar but the experimental and analytical curves almost coincide. Here, it is important to note that $0.53<\beta <0.878$ so that the scale exponent for the data collected corresponds to a sublinear behavior, without the blow-up phenomenon, in contrast to human cancers where the blow-up phenomenon is manifest as reported in Pérez-García et al. (2020). We believe that this result is probably a consequence of a limited amount of data or of the biological differences between humans and mice.

**Fig. 4:**
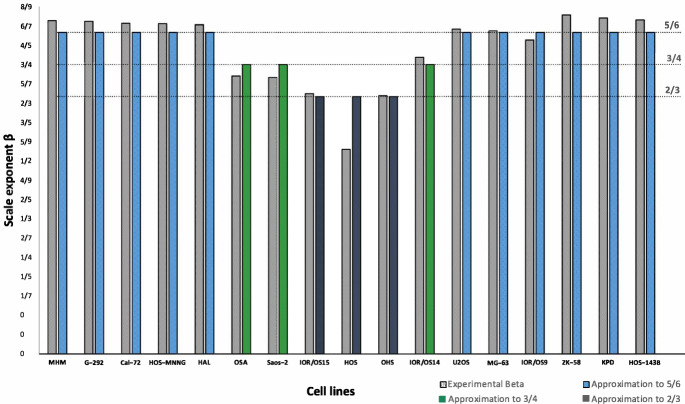
Distribution of the $\beta$ exponent for each cell line

The way in which cancer tumors grow corresponds to a multicellular spheroid with three layers. In the outer layer the proliferative cells are located, a little further inside is a layer in which the quiescent cells are located, and in a deeper layer the necrotic nucleus is created (Jones et al. 2009). According to Banavar et al. (2014), the two quantities that determine the metabolic scaling rate are the surface area and the rate of nutrient delivery or energy transport on the surface, and such distribution in our model is represented by the scaling exponent $\beta$. Also, we can observe that the cell lines tend to group around the values of exponent $\beta =2/3, 3/4, 5/6$, see Fig. 4.

The scaling exponent $\beta$ apparently reveals the fundamental physics underlying the relationship between geometry and physiology. For example, the exponent 2/3 could imply a smooth growth which seems to be due to a more complex circulation network with an intricate structure created by the tumor from the necrotic core to stay alive. For this reason dissipation occurs at the surface. An example of this behavior are the cell lines HOS, OHS and IOR/OS15.

On the other hand, the cell lines OSA, Saos-2 and IOR/OS14 have an exponent 3/4. It seems that in this case the internal structures together with the transport networks of nutrients and energy from the inner layers to the exteriors are simpler compared to the previous case.

Finally, with respect to the exponent 5/6 corresponding to the cell lines MHM, G-292, Cal-72, HOS-MNNG, HAL, U2OS, MG-63, IOR/OS9, ZK-58, KPD and HOS-143B, we can infer from the results that these cell lines have an inefficient transport structures with a low circulation.

When comparing the experimental results with the numerical results, the genetic complexity of the osteosarcoma cell lines becomes evident, however, the same results lead us to suggest the need to investigate what are the underlying biological and clinical aspects that each of them have in common when the cell lines are related to the same scale exponent.

### In Vitro Proliferation

**Fig. 5:**
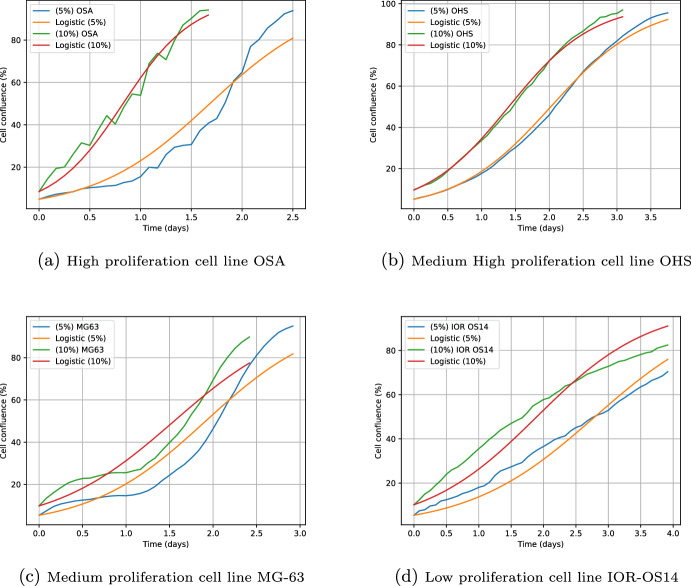
Logistic model approximations for four cell lines. The blue and green curves correspond to the $5\%$ and $10\%$ data of each cell line obtained from Lauvrak et al. (2013). The curves in yellow and red correspond to the approximations of the logistic model at $5\%$ and $10\%$ of each cell line

In this section we present the results obtained according to the model given in Sect. 2.3. In the same way as in Sect. 3.1, we obtained the values of the parameter $\rho$ in (4) which are shown in Table 2. There, cell lines are classified according to the values of $\rho$. This table shows the comparison in the values of the proliferation rates when the cells are seeded at $5 \%$ or $10 \%$ with respect to the load capacity of the Petri dish. Also, in Fig. 5 one can see the fit of some of the curves to the experimental data.

**Table 2:** Approximation of the $\rho$ parameters with logistic model

	Cell line	$\rho \mid _{5\%}$	$n \mid _{5\%}$	$R^2 \mid _{5\%}$	$\rho \mid _{10\%}$	$n \mid _{10\%}$	$R^2 \mid _{10\%}$	Weighted average
High	OSA	1.77	4	93.57%	2.88	2	97.33%	2.14
	MHM	1.75	3	96.81%	2.54	2	98.08%	2.07
	U2OS	1.92	4	98.92%	2.16	2	98.78%	2.00
	HOS-143B	1.77	6	97.52%	1.97	3	95.11%	1.84
	IOR/OS15	1.59	2	99.20%	1.89	2	96.61%	1.74
Medium high	Cal-72	1.45	3	98.44%	1.67	2	97.13%	1.54
	IOR/OS18	1.52	4	96.93%	1.54	2	96.98%	1.53
	IOR/OS10	1.48	6	97.38%	1.65	2	97.88%	1.53
	HOS	1.48	8	95.57%	1.58	2	95.76%	1.50
	OHS	1.44	7	99.63%	1.60	3	99.68%	1.49
Medium	IOR/SARG	1.32	3	99.57%	1.65	3	99.70%	1.48
	MG-63	1.50	8	92.35%	1.43	3	91.99%	1.48
	Saos-2	1.25	4	97.64%	1.86	2	97.05%	1.45
	HOS-MNNG	1.37	6	98.04%	1.43	3	97.19%	1.39
	ZK-58	1.17	2	98.56%	1.36	2	97.59%	1.26
Low	IOR OS14	1.02	3	95.23%	1.15	3	90.99%	1.09
	HAL	0.87	4	99.12%	1.22	4	99.61%	1.05
	IOR/OS9	0.93	6	99.36%	1.29	2	99.30%	1.02
	KPD	0.88	6	99.74%	1.00	3	99.42%	0.92
	IOR/MOS	0.78	2	99.64%	1.04	2	98.65%	0.91
	G-292	0.37	4	95.71%	0.43	2	93.50%	0.39

Ordered from highest to lowest according to the weighted average of the parameter $\rho$. *n* represents the number of the independent experiments

In the numerical results obtained for the values of rates $\rho$ in both scenarios, there are some differences in a range from $1\%$ to $63\%$. Among the lines that show the greatest change are OSA, Saos-2, MHM, HAL, IOR/OS9, IOR/MOS, IOR/OS15, ZK-58, see Table 2. Another important aspect is the order of the cell lines according to the values of $\rho$, which is similar to Lauvrak et al. (2013) for the cell proliferation. The differences present in the proliferation rates for each cell line could be attributed to the expression profiles of cell lines and the role of putative genes involved. This variability suggests the need to explore the underlying biological mechanisms that cause it. For example, in terms of the cell cycle, it would be interesting to investigate the regulation of oncogenes that make the cell duplication process more or less rapid, and whether the variations correspond to different degrees of sensitivity with respect to the passage number (the record of the number of times the cell line has been subcultured, i.e. harvested and reseeded into multiple vial “daughter” cell cultures (Freshney 2010)).

**Table 3:** Diffusion results

	Cell line	Times (days)	$\bar{D}$($\hbox {mm}^2$/day)	*sd*	CV
High	HOS	$<span class='convertEndash'>11-28</span>$	$1.19\times 10^{-2}$	$2.37 \times 10^{-3}$	$20\%$
	HOS-MNNG	$<span class='convertEndash'>8-21</span>$	$1.03 \times 10^{-2}$	$3.92\times 10^{-3}$	$38\%$
	OHS	$<span class='convertEndash'>3-28</span>$	$9.67\times 10^{-3}$	$7.44\times 10^{-4}$	$8\%$
	OSA	$<span class='convertEndash'>7-22</span>$	$9.26 \times 10^{-3}$	$9.88 \times 10^{-4}$	$11\%$
	HOS-143B	$<span class='convertEndash'>8-21</span>$	$8.35 \times 10^{-3}$	$3.55 \times 10^{-3}$	$42\%$
Medium high	HAL	$<span class='convertEndash'>7-28</span>$	$5.96 \times 10^{-3}$	$3.15 \times 10^{-3}$	$53\%$
	MHM	$<span class='convertEndash'>4-24</span>$	$5.92 \times 10^{-3}$	$2.68\times 10^{-3}$	$45\%$
	Cal$-72$	$<span class='convertEndash'>7-34</span>$	$3.95\times 10^{-3}$	$1.22 \times 10^{-3}$	$31\%$
	KPD	$<span class='convertEndash'>7-55</span>$	$3.33\times 10^{-3}$	$9.31\times 10^{-4}$	$28\%$
	G-292	$<span class='convertEndash'>18-69</span>$	$2.99 \times 10^{-3}$	$1.33\times 10^{-3}$	$44\%$
	IOR/OS9	$<span class='convertEndash'>16-48</span>$	$2.88\times 10^{-3}$	$1.89\times 10^{-3}$	$66\%$
	ZK$-58$	$<span class='convertEndash'>7-45</span>$	$1.66 \times 10^{-3}$	$4.54\times 10^{-4}$	$27\%$
Medium	Saos$-2$	$<span class='convertEndash'>15-90</span>$	$9.39 \times 10^{-4}$	$7.30 \times 10^{-5}$	$8\%$
	IOR/OS14	$<span class='convertEndash'>13-116</span>$	$5.55 \times 10^{-4}$	$1.78 \times 10^{-4}$	$32\%$
Low	IOR/OS15	$<span class='convertEndash'>11-129</span>$	$1.78\times 10^{-4}$	$4.41\times 10^{-5}$	$25\%$
	U20S	$<span class='convertEndash'>36-129</span>$	$1.34\times 10^{-4}$	$4.85\times 10^{-5}$	$36\%$
	MG$-63$	$<span class='convertEndash'>23-172</span>$	$5.01\times 10^{-5}$	$1.57\times 10^{-5}$	$31\%$

Ordered from highest to lowest according to the diffusion parameter ${\bar{D}}$. − Model does not apply, $+$ Model works

### Diffusion Process

For model (5), the value of the diffusion coefficient *D* is computed by considering the average in vitro proliferation rates $\rho$ of each cell line obtained in Sect. 3.2, as presented in Table 2. Table 3 shows the values of $\bar{D}$ (the average diffusion values during the experimental times). These findings facilitate the categorization of cell lines based on their diffusivity, ranging from high to low. Remarkably, the values of of $\bar{D}$ exhibit a similar order of magnitude as the diffusion coefficients reported for glioblastoma cells in Murray (2003).

**Fig. 6:**
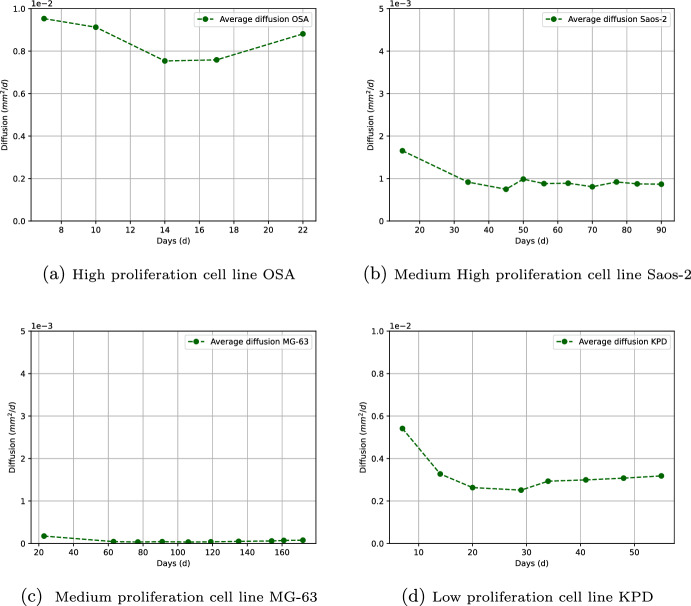
Diffusion for some cell lines with different levels of proliferation

Given that the number of data points is quite limited, we consider the criterion $\text {CV}<50\%$ for the coefficient of variation as a validation for the model. The lines marked in red ($12\%$) in Table 3 exhibit flaws in the model, while those marked in turquoise ($88\%$) indicate that the diffusion coefficient during the experimental measurement times remains close to a constant, see Fig. 6.

Although model (5) allows us to give a simple description of the diffusive behavior of the tumor, it certainly lacks many features inherent to this phenomenon, since, as explained in Serra-Picamal et al. (2012), this process involves the interaction of wave fronts affected by physiological and mechanical aspects which are neglected in the model.

For example, in Liotta et al. (1977) an explanation to this relationship was given for a field of transplanted host tissue with a fibrosarcoma. There, a model of diffusion of tumor growth vascularization and necrosis is proposed in order to describe the temporary changes in the radial distributions of cells tumors and blood vessels. The results obtained there show a maximum density of vessels at the front of tumor migration along with an increase in the maximum density of tumor cells which move away from the necrotic nucleus. That is, the dynamics of growth, the angiogenic and morphological factors are related to the movement of cells inside the tumor.

Studies as in Gerlee and Anderson (2010) present simulations and analysis of a hybrid cellular automaton model of tumor growth, highlighting the importance of the tumor microenvironment and in particular of oxygen concentrations for the morphology and for the dynamics of diffusion.

### Analysis of Results

In order to analyze our results we will compare the results for tumorigenicity, colony forming ability, invasion, migration, and proliferation shown in Table 3 in Lauvrak et al. (2013), where the cell lines are ordered according to their aggressiveness, with our results shown in Tables 1, 2, and 3. All these data are collected in Table 4. In this summary we use only the cellular lines for which all the characteristics are available.

**Table 4:** Summary of results $\alpha$, $\beta$, $\rho$, and $\bar{D}$ vs. experimental data

Cell line	$\alpha$	$\beta$	$\rho$	$\bar{D}$	TL	CL	IL	ML	PL
HOS	2.1797	0.53	1.5	$1.19 \times 10^{-2}$	4	508	1294	2406	3
OSA	1.1353	0.7198	2.14	$9.26 \times 10^{-3}$	4	465	678	2470	3
HOS-143B	0.8803	0.8649	1.84	$8.35 \times 10^{-3}$	4	376	1010	2048	3
HOS-MNNG	0.8598	0.8555	1.39	$1.03 \times 10^{-2}$	4	590	887	1513	2
MHM	0.8137	0.8633	2.07	$5.92 \times 10^{-3}$	4	177	580	888	3
OHS	1.164	0.6696	1.49	$9.67 \times 10^{-3}$	4	56	195	299	2
Cal-72	0.3021	0.8562	1.54	$3.95 \times 10^{-3}$	3	230	41	246	2
HAL	0.4663	0.853	1.05	$5.96 \times 10^{-3}$	3	3	1045	1863	1
IOR/OS9	0.4351	0.8134	1.02	$2.88 \times 10^{-3}$	3	9	108	347	1
ZK-58	0.3742	0.8781	1.26	$1.66 \times 10^{-3}$	3	2	4	127	2
KPD	0.1668	0.8704	0.92	$3.33 \times 10^{-3}$	2	240	90	291	1
G-292	0.2016	0.8619	0.39	$2.99 \times 10^{-3}$	2	242	32	156	1
Saos-2	0.2573	0.7161	1.45	$9.39 \times 10^{-4}$	2	52	109	697	2
IOR/OS14	0.1576	0.7687	1.09	$5.55 \times 10^{-4}$	2	8	109	507	2
MG-63	0.0801	0.8377	1.48	$5.01 \times 10^{-5}$	1	695	2	31	3
IOR/OS15	0.1251	0.6742	1.74	$1.78 \times 10^{-4}$	1	94	467	931	3
U2OS	0.1	0.8419	2.00	$1.34 \times 10^{-4}$	1	19	771	2039	3

*Data for TL *Tumorigenicity, *CL *Colony forming ability, *IL *Invasion, *ML* Migration, *PL *Proliferation was taken Table 3 from Lauvrak et al. (2013)

**Fig. 7:**
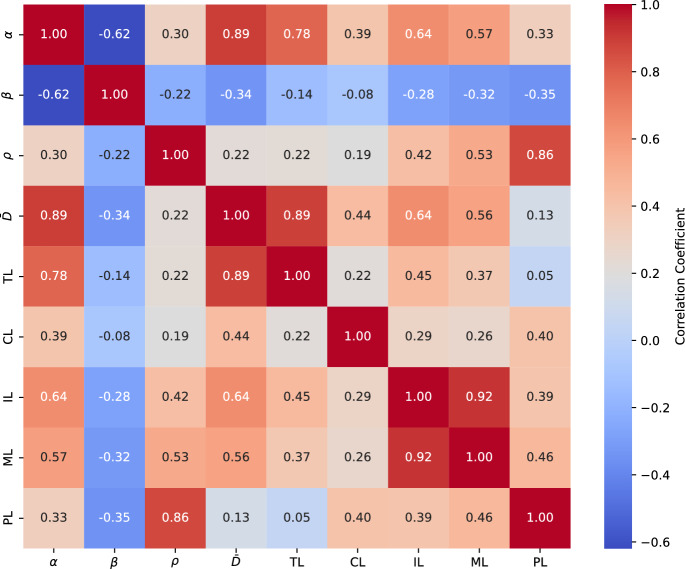
Correlation Matrix. *TL *Tumorigenicity, *CL *Colony forming ability, *IL *Invasion, *ML* Migration, *PL *Proliferation

With the data presented in Table 4 we generated the correlation matrix given in Fig. 7 between the values of growth rate ($\alpha$), scale exponent ($\beta$), logistic parameter ($\rho$), diffusion coefficient (${\bar{D}}$) and the values taking from Lauvrak et al. (2013) for tumorigenicity (TL), colony forming ability (CL), invasion (IL), migration (ML) and proliferation (PL).

To describe tumor growth, we employed the power law, which helped elucidate the behavior of cancerous tumor growth in immunosuppressed mice, as observed in Sect. 3.1. The values of the parameter $\alpha$ in model (1) determine the growth rates of the tumor volume, that is, the rate at which the tumor grows over time, and therefore the aggressiveness of each cell line, which correlates directly with the classification for tumorigenicity obtained in Lauvrak et al. (2013). It would be interesting to explore experimentally the relation between $\alpha$ and tumorigenicity based on the hypothesis that the higher value of tumor growth rate could imply a higher tumorigenicity.

In our statistical analysis, we note that the correlation coefficients between the parameter $\alpha$, representing the tumor growth rate, the diffusion coefficients $\bar{D}$, and the tumorigenicity obtained experimentally are quite high. Also, the correlation between $\alpha$ and invasion caracteristics from Lauvrak et al. (2013) is moderately high. To describe how these processes are interconnected would also mean to describe the relationship between the motility of the tumor cells and their ability to migrate, survive and proliferate in environments different from the original tumor, as well as their ability to evade the immune system and promote tumor growth, Fidler (2003), Friedl and Wolf (2003); it would be interesting to investigate these relations in the future.

Table 4 shows that the most aggressive behavior is exhibited by tumors associated with the cell lines HOS, HOS-MNNG, OSA, and HOS-143B, which coincides with the conclusions, for example, in Botter et al. (2015).

While numerous studies have utilized various cell lines, detailed information regarding their origins and characteristics is often limited. To facilitate interpretation of our findings, we provide a brief overview of several cell lines:

The HOS cell line, established by McAllister et al. in 1971 from a tumor of a 13-year-old girl (McAllister et al. 1971), exhibits a flat morphology, low saturation density, low plating efficiency in soft agar, and sensitivity to chemical and viral transformation [American Type Culture Collection (ATCC)]. In Lauvrak et al. (2013), it is classified as one of the most aggressive cell lines. In our results, HOS cells display high values for $\alpha$ and $\bar{D}$ and a medium high value for $\rho$.

HOS-143B is a cell line derived from the HOS line, the cells of this line are a suitable transfection host [American Type Culture Collection (ATCC)]. This cell line is an exceptional case in our study as it shows high levels in all analyzed categories. In Mohseny et al. (2011), it is stated that this cell line stands out for its robust metastatic potential, making it an important line in the study of metastasis in humans, which is a widespread clinical problem in osteosarcoma patients.

Another line showing interesting behavior is the OSA line, as it exhibits high values of $\alpha$, $\rho$, $\bar{D}$, TL, CL, IL, ML, PL. This line was established in 1982, has a fibroblastic morphology, and originates from a 19-year-old black man diagnosed with primitive multipotential sarcoma of the femur (American Type Culture Collection (ATCC)). According to Ottaviano et al. (2010), it was possible to determine that this cell line presents high levels of MDM2 proteins and positive stains for TP53; the combination of these two factors predisposes to high tumor growth.

The HOS-MNNG cell line, a derivative of the HOS line generated via transformation with the carcinogenic nitrosamine MNNG at a concentration of 0.01 mcg/ml, demonstrates high saturation density, robust soft agar colony formation, and tumorigenicity in nude mice (American Type Culture Collection (ATCC)). These findings are consistent with those reported by Mohseny et al. (2011) and Lauvrak et al. (2013). Our models indicate that HOS-MNNG cells exhibit elevated values for $\alpha$ and $\bar{D}$ in accordance with experimental observations for tumorigenicity presenting meanwhile a medium value for $\rho$.

The MHM cell line originated from an osteosarcoma of pelvic bones of a 41-year-old woman and presents a fibroblastic morphology (Bairoch). According to our results, this line exhibits high values of $\alpha$ and tumorigenicity confirming again that these characteristics are interrelated. It is interesting to note that in Mohseny et al. (2011) it is observed that the cell duplication is not too rapid, and its capacity to invade adjacent tissues without metastases is confirmed.

The OHS cell line was established from a secondary primary cancer in a 14-year-old caucasian boy (Ottaviano et al. 2010). According to Fodstad et al. (1986), a key characteristic of the OHS cell line is its remarkable similarity to the cultured and biopsied cells, with minimal morphological differences observed. This feature makes it a valuable model for elucidating the genetic factors that predispose individuals to osteosarcoma, particularly in the context of retinoblastoma, given the shared genetic characteristics of these two cancers, including *RB* gene mutations (Hansen et al. 1985). For this line, our models predict high values for $\alpha$ and $\bar{D}$, but a medium high value for $\rho$. While Lauvrak et al. (2013) reports high tumorigenicity for this cell line, other characteristics exhibit medium values.

The Cal-72 cell line, derived from a knee osteosarcoma in a 10-year-old child, exhibits osteoblastic characteristics based on morphological, immunohistochemical, and molecular analyses (Rochet et al. 1999); this study also elucidates the role of HGF/scatter factor in modulating the motility of these cells. Our findings demonstrate a medium high value for $\alpha$ and $\bar{D}$ in concordance with the results for tumorigenicity in Lauvrak et al. (2013).

The HAL cell line was derived from an osteosarcoma in a 16-year-old male (Bairoch). According to our models, it is classified as a low-proliferation line with medium high values for both $\alpha$ as $\bar{D}$. While these results align with Lauvrak et al. (2013) regarding tumorigenicity, they contrast with the findings reported by Mohseny et al. (2011), where this cell line did not exhibit tumor growth in vivo or invasive behavior. However, Ottaviano et al. (2010) reports the presence of CDKN2A in HAL cells, a gene whose mutations can lead to rapid and uncontrolled cell division or inhibit apoptosis, as described in [National Cancer Institute (NCI)], potentially corroborating the observations of Lauvrak et al. (2013).

The ZK-58 cell line was derived from a 21-year-old male and exhibits an osteoblastic phenotype, as reported in Ottaviano et al. (2010), where CDKN2A protein expression is 100$\%$. According to Mohseny et al. (2011), in vitro differentiation confirms an osteosarcoma origin, although the cell line did not exhibit tumorigenic behavior. However, for this line (Lauvrak et al. 2013) reported a medium high tumorigenicity, and we obtained the values for $\alpha$ and $\bar{D}$ in the same category.

As for the G-292 cell line, it is known that it comes from a 9-year-old white female and presents a fibroblastic morphology (American Type Culture Collection (ATCC)). According to Table 4 this line presents the values of $\alpha$, $\rho$ and ${\bar{D}}$ in different categories. We were unable to find more information.

The Saos-2 line was obtained from the primary osteosarcoma of an 11-year-old girl as a part of an extensive series of human tumor cell lines isolated and characterized by J. Fogh and G. Trempe as reported in (American Type Culture Collection (ATCC)). This line exhibits epithelial morphology and these cells can fully differentiate in the same way as osteoblast cells do (Hausser and Brenner 2005). Although this line has been widely used as a model for the human osteoblast cell system, its phenotypic stability has not been definitively determined. According to Hausser and Brenner (2005), the passage number affects proliferation rates, with higher passage cells exhibiting increased proliferation. Therefore, we hypothesize that the observed differences in proliferation rates at 5% and 10% may be attributed to this phenomenon. Saos-2 cells exhibit medium values for $\alpha$, $\bar{D}$ and tumorigenicity.

The MG-63 cell line, derived from a 14-year-old male with osteosarcoma (Merck KGaA (Merck) 2023), exhibits an immature osteoblastic phenotype characterized by rapid proliferation and lack of contact inhibition, as reported by Czekanska et al. (2012). In vivo studies conducted by Ottaviano et al. (2010) did not demonstrate tumorigenic or invasive potential for this cell line, corroborating the findings of Lauvrak et al. (2013) and our own calculations.

The IOR/OS15 cell line is derived from a 12-year-old girl (Bairoch). This osteoblastic cell line has shown no evidence of tumorigenicity, invasion, angiogenesis, or metastasis as reported by Mohseny et al. (2011), consistent with Lauvrak et al. (2013) and our modeling results. The discrepancy between its high proliferation rate and relatively low diffusion rate is noteworthy.

The U2OS cell line, established by Pontén and Saksela in 1964 (Pontén and Saksela 1967), from a moderately differentiated sarcoma of the tibia in a 15-year-old girl (Merck KGaA (Merck)), represents one of the first human cell lines derived from a mesenchymal tumor. According to Lauvrak et al. (2013) and our numerical results, U2OS exhibits high values of $\rho$ but low values for $\alpha$, ${\bar{D}}$ and TL. This cell line exhibits similarities to platelet-derived growth factor (PDGF), as reported by European Collection of Authenticated Cell Cultures (ECACC) (2017).

In general, it could be expected that tumors with faster growth rates would demonstrate greater diffusion capacity and higher tumorigenicity, leading to more aggressive and potentially more dangerous outcomes. However, the precise relationship between these factors likely varies depending on the specific tumor type, its microenvironment, and a multitude of other biological and genetic determinants.

Regarding cell proliferation, the use of population models with sigmoid-type solutions allows us to obtain a good approximation to the proliferation rates, as validated by the results obtained in Sect. 3.2, where a good approximation to the experimental data was achieved with a correlation coefficient of 0.86.

On the other hand, the scale exponent $\beta$ reveals interesting aspects of the underlying behavior of tumor growth, such as sublinear tumor volume variation without the existence of explosive behavior. Furthermore, the grouping of scale exponent values around 2/3, 3/4, and 5/6 suggests the need to further explore biological aspects such as the relationship between metabolic rate and tumor volume. According to the correlation matrix, the parameter $\beta$ correlates weakly with the other variables.

## Conclusions

The models used to describe tumor growth and cell proliferation of the analyzed osteosarcoma cell lines turned out to be effective. The diffusion model showed that the diffusion coefficient of osteosarcoma tends to exhibit a constant behavior, an idea also noted by Murray (2003) for glioblastoma.

For tumor growth, the parameters $\alpha$ and $\beta$ of the proposed model were adjusted using experimental volume tumor data through the least squares method. The parameter $\alpha$ is interpreted as a potential tumor growth rate, which exhibits sublinearity in growth contrasted with the blow-up phenomenon observed in human cancers.

Determining the in vitro proliferation coefficients of cell lines is crucial for understanding the behavior of cancerous tumors and for making clinical prognoses. Despite the genetic complexity of osteosarcoma, using logistic models and analysis of experimental data, we approximated cell proliferation rates for various cell lines.

The exponential, logistic, Gompertz, Bertalanffy, and other models have been widely used to describe the proliferation of cancerous cells. Determining the in vitro proliferation coefficients of cell lines is crucial for understanding the behavior of cancerous tumors and for making clinical prognoses. Despite the genetic complexity of osteosarcoma, using logistic models and analyzing experimental data, we approximated cell proliferation rates for various cell lines. Recently, in Wahbi et al. (2024), this type of ordinary differential equations was used to describe tumor cell proliferation and their kinetic behavior under inhibitory treatments. It was also shown that the tumor growth of some human cancers exhibits a scaling coefficient of 2/3, which was one of the scaling exponents observed in some of the osteosarcoma cell lines studied in our work (IOR/OS15, HOS, OHS, see Fig. 4). Therefore, we consider it important to explore which types of human cancers exhibit the scaling exponents 2/3, 3/4, and 5/6, as observed in some of the osteosarcoma cell lines studied in the present work.

According to numerical experiments, a diversity in proliferation rates among different cell lines can be observed, indicating variability in the aggressiveness of each one of them. These differences may be related to specific gene expression profiles and regulation of genes related to oncogenesis. An important aspect to highlight is the observation that proliferation rates vary depending on cell seeding density and the capacity of the culture plate, suggesting sensitivity to growth conditions or passage number. These differences suggest the need to investigate the underlying biological mechanisms that produce them. For example, studying the regulation of the cell cycle and the impact of oncogenes on cell duplication processes could provide a deeper understanding of the observed discrepancies.

Studying the cellular diffusion process in the context of osteosarcoma, along with describing the speed of tumor growth and proliferation, is fundamental for understanding tumor biology and developing effective therapeutic strategies. Cell diffusion, guided by extracellular chemical gradients, plays a crucial role in the invasion and metastasis of cancer cells. Using experimental data from in vivo tumor growth and in vitro cell proliferation, we applied the model from Murray (2003), which provides a spatiotemporal analysis of osteosarcoma cell lines.

To use this model, we assumed an initial centrifugal tumor growth and exponential cell reproduction. The explicit solution of the model allows us to calculate the diffusion coefficient. The experimental data reveal a wide variability in the times of the tumor appearance, highlighting the variability in osteosarcoma behavior.

Although the model used is quite simple, reducing physiological and mechanical aspects of tumor diffusion to its minimum, our numerical results show significant consistency with experimental data for tumorigenicity.

It is interesting to note that the correlation coefficients between our numerical results for tumor growth rate $\alpha$, diffusion coefficients $\bar{D}$, and experimentally described tumorigenicity in Lauvrak et al. (2013) are high. As Semenza (2012) suggests, the hypoxic conditions activate transcription of the genes which transport glucose and glycolytic enzimes and thus boost the Warburg effect. On the other hand, the results obtained in Gerlee and Anderson (2010) show how oxygen concentration appears to be related to the impact of diffusion on tumor growth. This suggests that in the laboratory-analyzed osteosarcoma cell lines, rapid tumor growth may be related to increased diffusion capacity.

The numerically obtained proliferation coefficients $\rho$ and the experimentally given proliferation data have a correlation coefficient of 0.86. On the other hand, the correlation coefficients between proliferation, tumor growth, diffusion, tumorigenicity, invasion, migration and colony-forming ability turn out to be weak.

Despite the simplicity of the models used, an adequate description of in vivo tumorigenicity and in vitro proliferation was achieved, allowing us to approximate the diffusive behavior of osteosarcoma. The order of the diffusion coefficients of osteosarcoma and those presented in Murray (2003) is the same.

In Santra and Samanta (2024), numerical simulations of a partial differential equation system considering tumor, hunter, and resting cells identified various spatial distributions as a function of the parameters posed in the problem. Using a reaction-diffusion model, the study explores the spatial aspects of the interaction between the immune system and tumors, analyzing how spatial heterogeneity can influence both tumor growth and the immune response. Although the approach used in our study to describe diffusive behavior differs from that adopted in Santra and Samanta (2024), both studies theoretically demonstrate differences in the diffusive behavior of tumors. However, in our case, there appears a close relationship between the diffusion coefficient $\bar{D}$ and the tumor volume growth parameter $\alpha$, as shown in Fig. 7. Moreover, ${\bar{D}}$ has a moderate positive correlation with the experimental data reported by Lauvrak et al. (2013) for migration and invasion. In Santra and Samanta (2024), on the other hand, tumor cells exhibit variations in their propagation depending on different tissue environments. As stated in that paper, these results suggest that the spatial behavior of cancerous cells could be a key factor in designing more effective therapeutic strategies.

The model presented in Le et al. (2021) does not include spatial distributions and uses the parameter $\lambda$ to represent the reproduction rate in chemical reactions between different states. The data in Table 1 from our study show the values of $\alpha$ (a parameter measuring changes in tumor growth) and $\beta$ (a scaling exponent parameter), while Table 2 presents the values of the logistic parameter $\rho$. These tables highlight differences in tumor growth and proliferation behavior depending on the cell line considered. In Le et al. (2021), the parameters were estimated for each group of patients with different immune compositions, allowing for the identification of variations in prognoses and treatment responses. Exploring the relationship between immune compositions and cell lines would be relevant, as observed in Tables 1 and 2 of our study, where the cell lines induce specific changes in both tumor volume and proliferation rates. Although the proliferation coefficient $\lambda$ in Le et al. (2021) is applied in a different context to our parameter $\rho$, comparing these parameters could be useful from a mathematical perspective to determine if their orders are similar and to investigate the biochemical processes associated with the cell lines described. This comparison could open new avenues in both mathematical modeling and clinical research, motivating further studies on this topic.

The findings obtained with these models suggest the need for specific clinical studies to more accurately determine the role of diffusion in the survival of patients with osteosarcoma and to explore how diffusion and proliferation processes influence tumor growth and invasion. Such research could provide information for the development of new medical strategies aimed at blocking diffusion and tumor growth in osteosarcoma.

## Data Availability

Experimental data corresponding to the variation of tumor volume several days after injection for each cell line in in vivo studies and the ability of each cell line to cover the Petri dish in hours with initial conditions at 5% and 10% in in vitro studies, are publicly available and can be found in the supplementary material (Lauvrak et al. 2013).

## Footnotes

1 See: https://docs.scipy.org/doc/scipy/reference/generated/scipy.optimize.curve_fit.html, January 2023.
